# Quantitative and dynamic measurements of biological fresh samples with X-ray phase contrast tomography

**DOI:** 10.1107/S1600577514018128

**Published:** 2014-10-08

**Authors:** Masato Hoshino, Kentaro Uesugi, Takuro Tsukube, Naoto Yagi

**Affiliations:** a Research and Utilization Division, Japan Synchrotron Radiation Research Institute, 1-1-1 Kouto, Sayo, Hyogo 679-5198, Japan; b Japanese Red Cross Kobe Hospital, 1-3-1 Wakinohamakaigandori, Chuo-ku, Kobe, Hyogo 651-0073, Japan

**Keywords:** X-ray phase contrast tomography, quantitative imaging, three-dimensional densitometry, dynamic imaging

## Abstract

X-ray phase contrast tomography using a Talbot grating interferometer was applied to biological fresh samples which were not fixed by any fixatives. To achieve a high-throughput measurement for the fresh samples the X-ray phase contrast tomography measurement procedure was improved. The three-dimensional structure of a fresh mouse fetus was clearly depicted as a mass density map using X-ray phase contrast tomography. The mouse fetus measured in the fresh state was then fixed by formalin and measured in the fixed state. The influence of the formalin fixation on soft tissue was quantitatively evaluated by comparing the fresh and fixed samples. X-ray phase contrast tomography was also applied to the dynamic measurement of a biological fresh sample. Morphological changes of a ring-shaped fresh pig aorta were measured tomographically under different degrees of stretching.

## Abstract 2

Quantitative measurements of biological fresh samples based on three-dimensional densitometry using X-ray phase contrast tomography are presented.

## 1. Introduction   

The high penetration power of X-rays is useful for measuring the inner structures of opaque samples without destruction. On the other hand, biological soft tissues, which are mainly composed of light elements (hydrogen, oxygen, carbon and so on), are almost transparent to hard X-rays. Thus, absorption-based imaging typically produces poor image contrast for soft tissues. To improve the image contrast, X-ray phase contrast imaging techniques have been developed (Bonse & Hart, 1965 ▶; Momose *et al.*, 1995 ▶; Davis *et al.*, 1995 ▶; Snigirev *et al.*, 1995 ▶; Schmahl *et al.*, 1995 ▶; Wilkins *et al.*, 1996 ▶; Chapman *et al.*, 1997 ▶). In general, the complex refractive index of X-rays is represented as *n* = 1 − δ + *i*β. The phase factor δ is approximately 10^3^ times higher than the absorption factor β for light elements in the hard X-ray region (Momose & Fukuda, 1995 ▶). This means that phase contrast imaging using δ has a much higher sensitivity for soft tissue compared with simple absorption contrast imaging expressed as a function of β. High sensitivity in phase contrast imaging is suitable for visualizing small density differences which often represent important structural information in biological soft tissue.

X-ray Talbot grating interferometry, which was first demonstrated by Momose *et al.* using a synchrotron light source, is one of the phase contrast imaging techniques (Momose *et al.*, 2003 ▶; Weitkamp *et al.*, 2005 ▶). With this technique a differential phase shift can be measured from disturbances of the wavefront caused by penetration through a sample. This grating interferometer is only composed of two transmission gratings under quasi-parallel beam illumination such as by synchrotron radiation. Although several X-ray interferometric techniques have been developed, the grating interferometer technnnique has outstanding features compared with other techniques. For instance, a density resolution of around 1 mg cm^−3^ is achievable in X-ray phase contrast tomography and the optical setup is very simple as pointed out previously (Momose *et al.*, 2006 ▶). The high sensitivity makes it possible to visualize small density differences in soft tissue with sufficient image contrast. From this perspective, this imaging technique is a powerful tool for biomedical research. Several applications of the grating interferometer technique based on synchrotron radiation have already been reported. A rat whole brain, which is regarded as absolute soft tissue, has been clearly visualized by X-ray phase contrast tomography using a grating interferometer (Pfeiffer *et al.*, 2007*a* ▶; McDonald *et al.*, 2009 ▶). In the case of a human sample, Purkinje cells in the human cerebellum could be measured by high-spatial-resolution X-ray phase contrast tomography (Schulz *et al.*, 2010*a* ▶). The carotid artery and vein in a mouse were measured under *in situ* conditions with much higher image contrast compared with absorption-based imaging (Xi *et al.*, 2012 ▶). Moreover, X-ray phase contrast tomography based on a conventional laboratory source has also been applied to the measurement of biological soft tissue (Pfeiffer *et al.*, 2007*b* ▶; Bech *et al.*, 2009 ▶).

At SPring-8, an X-ray phase contrast tomography system using a grating interferometer has been developed (Hoshino *et al.*, 2012*a* ▶). So far this imaging system has been applied to several biological samples. The three-dimensional structure of various organs in a formalin-fixed mouse fetus could be clearly measured (Hoshino *et al.*, 2012*b* ▶). This information would be useful for examining abnormalities during development. This technique has also been applied to a quantitative analysis of crystalline concentration in eye lenses (Hoshino *et al.*, 2011 ▶). In both cases it was difficult to obtain adequate image contrast by simple absorption contrast imaging.

In our previous system with a step-by-step high-resolution scan, it was only possible to obtain high-quality X-ray phase contrast images from formalin-fixed samples. However, this sometimes limited the possible applications. To extend the range of applications of X-ray phase contrast imaging and tomography, a more flexible system was required. One interesting application is the measurement of fresh samples that are not fixed by any fixative. Pfeiffer *et al.* reported the validity of X-ray phase contrast tomography for application to non-fixed biological soft tissue (Pfeiffer *et al.*, 2009 ▶). Phase contrast images of fresh samples would reveal the original density map which is not affected by any fixatives. More accurate and quantitative information could be obtained from them. Another interesting application is dynamic measurement of a fresh sample. A quantitative analysis is currently limited to static samples. A new aspect of the sample may become available if the quantitative analysis using X-ray phase contrast tomography is applicable to dynamic measurements. These scientific cases require a high-throughput imaging system while high image quality should be maintained.

In this report a high-throughput X-ray phase contrast tomography system using a grating interferometer and its application to high-sensitive and quantitative measurements of biological fresh samples are presented. Results show a quantitative evaluation of the effect of a fixative on biological soft tissues by direct comparison of the fresh and fixed samples. In addition, a preliminary experimental result of the application to a quantitative dynamic measurement of a fresh sample using X-ray phase contrast tomography is also presented.

## 2. High-throughput X-ray phase contrast tomography using the Talbot grating interferometer   

### 2.1. X-ray Talbot grating interferometer   

**Figure 1:**
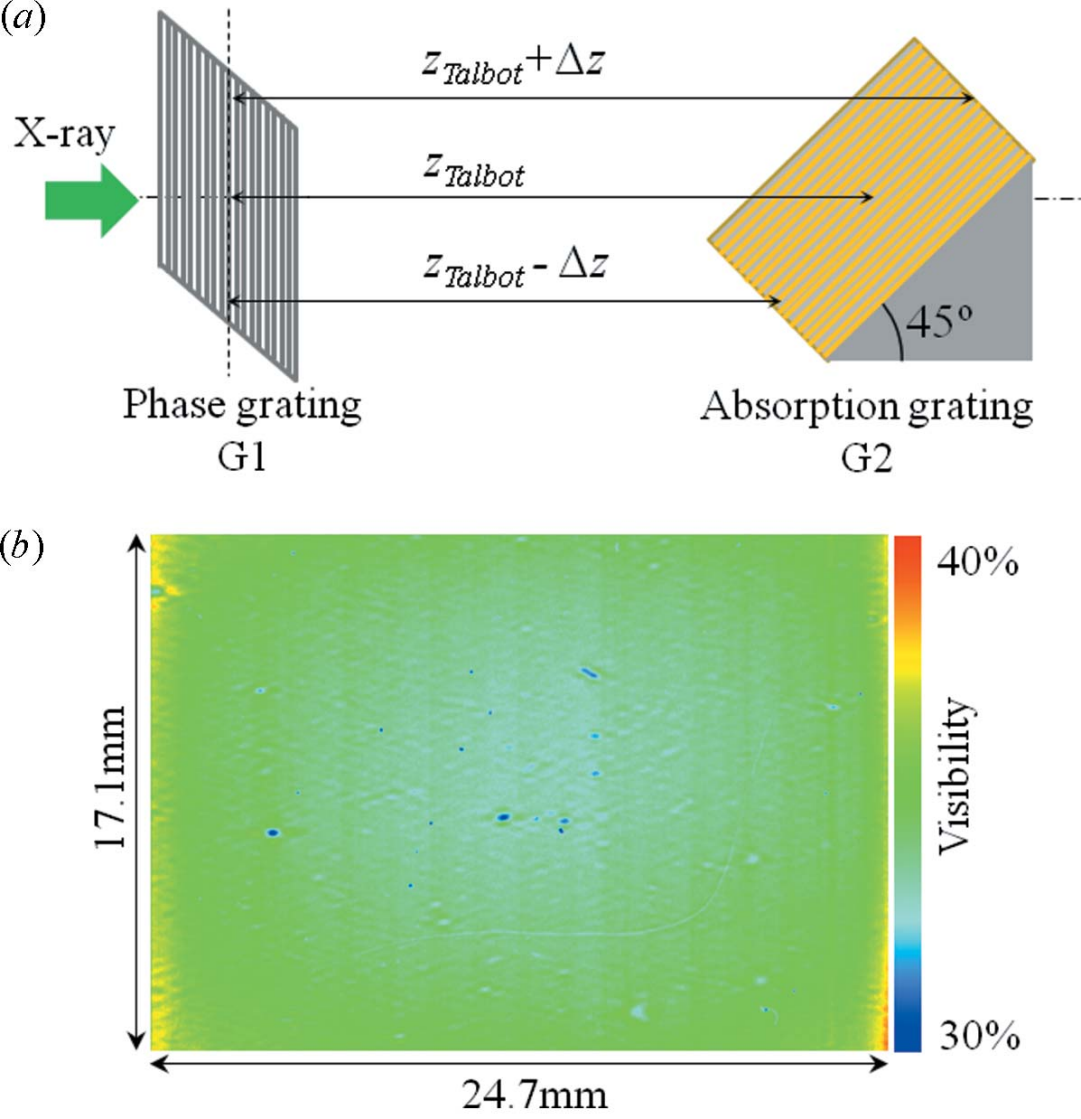
(*a*) Schematic drawing of the relation between G1 and G2. The difference in the Talbot distance caused by inclination of G2 is expressed as Δ*z*. (*b*) Visibility map of Moiré fringes in the effective field of view of the grating interferometer.

An X-ray phase contrast imaging system is based on the Talbot grating interferometer. A grating interferometer has been constructed at the bending magnet beamline BL20B2 at SPring-8 (Goto *et al.*, 2001 ▶). The experimental station is located at more than 200 m from the source. A monochromatic X-ray beam with a large cross section, approximately 300 mm (H) × 20 mm (V), is available at the station. The grating interferometer consists of a phase grating (G1) and an absorption grating (G2). G1 is set just behind the sample; G2 is set at the third-order fractional Talbot distance of G1 where a self image of G1 is created. G1 is made of tantalum with a pattern thickness of 2.1 µm; G2 is made of gold with a pattern thickness of 16.6 µm. As shown in Fig. 1(*a*) ▶, the G2 pattern is inclined towards the optical axis to increase the effective absorption by the grating. The grating pattern is drawn on a 4-inch silicon wafer by electron beam lithography (fabricated by NTT-AT, Japan). The grating pitch and area size in both gratings are 10 µm and 25 mm × 25 mm, respectively. In the case of the G1 used in this system, the optimum X-ray energy which produces a phase shift of π/2 when penetrating the grating pattern is 18.8 keV. With this optimum X-ray energy, maximum visibility on the Moiré fringe would be obtained. In the actual measurement, however, an X-ray energy of 25 keV was used to secure the X-ray transmission through a water cell in which a biological sample is immersed during the measurement. Although there is a reduction in the visibility of Moiré fringes due to the use of a higher than optimum X-ray energy, more than 30% visibility is obtained at the third-order Talbot distance (Hoshino *et al.*, 2012*a* ▶). A vacuum path with Kapton windows is set between G1 and G2 to avoid absorption and scattering by air.

By inclining G2, the distance between G1 and G2, which is defined as the Talbot distance, varies between the upper and lower parts in the effective field of view of the interferometer as shown in Fig. 1(*a*) ▶. The Talbot distance when using a phase grating is defined as
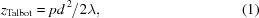
where *d* is the grating pitch, λ is the X-ray wavelength and *p* is the Talbot order (in this case *p* is an odd number, *p* = 1, 3, 5,…). Under the current experimental conditions shown above, *z*_Talbot_ is calculated to be 3024 mm. Since the Talbot distance is a function of the X-ray wavelength, the difference in the distance Δ*z* from *z*_Talbot_ caused by the inclination of G2 can be discussed by considering the virtual shift of X-ray wavelength Δλ from the central wavelength λ. The maximum value of Δ*z* when the centre of G2 is set at *z*_Talbot_ is Δ*z* = 8.84 mm at an inclination angle of 45°. Under this condition the virtual shift of wavelength when the G1–G2 distance becomes *z*_Talbot_ ± Δ*z* is Δλ = 2.9 × 10^−4^ nm. Here, Δλ/λ is calculated to be 5.8 × 10^−3^. This is much smaller than the limit of achromaticity in the grating interferometer (Weitkamp *et al.*, 2005 ▶). This means that the inclination of G2 has no effect on the phase contrast imaging. Actually, the Moiré fringes in the effective field of view have almost uniform visibility as shown in Fig. 1(*b*) ▶.

A sample is suspended by a specially designed sample holder and immersed into a water cell filled with a normal saline (Hoshino *et al.*, 2011 ▶, 2012*b* ▶). The sample is rotated in the water cell in order to carry out X-ray phase contrast tomography measurements. The thickness of the water cell is 25 mm so that it should be equal to the width of the grating area. This water cell enables samples with diameters up to the width of the grating area to be measured using X-ray phase contrast tomography. The X-ray transmission for the water cell when the X-ray energy is 25 keV is 34% while the X-ray transmission at 18.8 keV is 13%.

The X-ray detector is a visible-light conversion type imaging detector composed of a beam monitor (BM5; Hamamatsu Photonics, Japan) and a scientific CMOS camera ORCA Flash 4.0 (2048 × 2048 pixel format, 6.5 µm pixel size; Hamamatsu Photonics, Japan) (Uesugi *et al.*, 2011*a* ▶, 2012 ▶). The incident X-ray beam onto BM5 is converted into visible light by a P43 powder (Gd_2_O_2_S:Tb^+^) phosphor screen of thickness 25 µm or 50 µm. Although a thicker phosphor screen is useful for efficient measurement, the achievable spatial resolution is limited by the phosphor, which is approximately 30 µm (Uesugi *et al.*, 2011*a* ▶). The visible-light image is focused onto the sCMOS device using a lens system. The lens system consists of a large-aperture lens with a focal length of 200 mm and a camera lens with a focal length of 85 mm (Nikon, AF-S Nikkor 85 mm f/1.4G). Since the magnification factor of the lens system is 0.425×, the effective pixel size of the X-ray detector is equal to 15.5 µm. Then the effective field of view of the X-ray detector corresponds to 31.7 mm (H) × 31.7 mm (V). The field of view of the phase contrast imaging system is 24.7 mm (H) × 17.1 mm (V), which is limited by the grating area size. The acquisition timing can be controlled by the external TTL trigger signals.

### 2.2. High-throughput X-ray phase contrast tomography   

In the case of X-ray phase contrast imaging and tomography based on the interferometric technique, a phase retrieval process must be considered. In general, two kinds of phase retrieval techniques are available for the grating interferometer. A ‘single shot’ Fourier transform method is useful for a fast measurement since it is easy to control the Moiré fringes. Four-dimensional X-ray phase contrast tomography has been demonstrated using this method and white synchrotron radiation (Momose *et al.*, 2011 ▶). However, the spatial resolution of the phase image is restricted by the period of the carrier fringes. Fine carrier fringes also reduce the visibility on the detector due to the modulation transfer function of the pixel array. To obtain high spatial resolution and high sensitivity, a phase-stepping method is more appropriate. In this case it is important to complete the phase-stepping procedure quickly in order to achieve a high-throughput measurement. Efficient scanning methods for the phase stepping such as an interlaced method have been proposed (Zanette *et al.*, 2011 ▶, 2012 ▶). In the present study a high-throughput system using the phase-stepping method was developed to measure a fresh sample.

**Figure 2:**
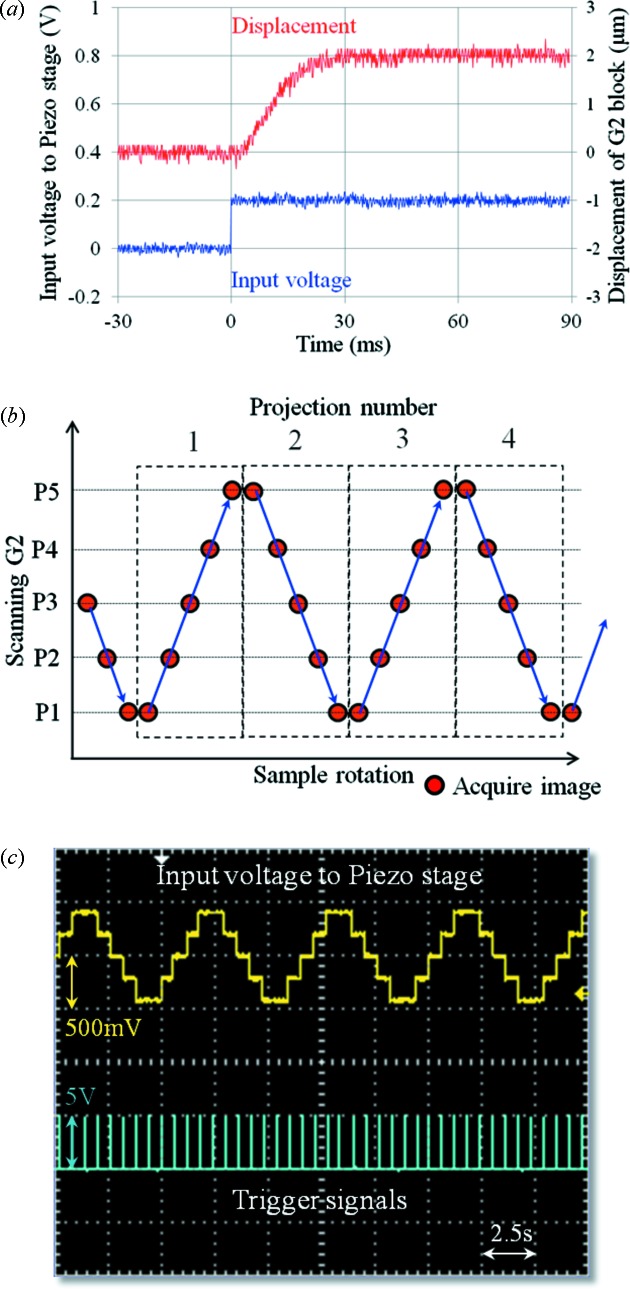
(*a*) Response of the piezo stage used for scanning G2. The displacement of the G2 block and input voltage to the piezo stage were measured using a TDS2004C oscilloscope. (*b*) Schematic diagram of the scanning procedure used in X-ray phase contrast tomography. (*c*) Actual input voltage signals and trigger signals during measurement (display on the TDS2004C oscilloscope).

A large piezo stage (P-733.2CL; Physik Instrumente) was introduced to scan G2 repetitively whereas an accurate stepping motor stage was used in the previous system. The response of the piezo stage when the G2 block was loaded onto the stage was measured in order to determine the scanning conditions. The G2 block consists of G2 with a specific holder and an aluminium block to incline the grating. The total weight of the G2 block was approximately 800 g. The piezo stage was operated under closed-loop conditions. Displacement of the G2 block was measured using a laser displacement meter (LK-H008; KEYENCE). A multi-function data acquisition system (USB-6211; National Instruments) was used to control the piezo stage. The input voltage and displacement of the G2 block were measured using a digital oscilloscope (TDS2004C; Tektronix). The result is shown in Fig. 2(*a*) ▶. It took approximately 30 ms to complete the movement after receiving the signal.

The scanning procedure in X-ray phase contrast tomography was improved to achieve efficient measurements. A schematic diagram of the scanning procedure is shown in Fig. 2(*b*) ▶. The horizontal axis represents the rotation of a sample. This diagram shows that the sample is continuously rotating during five-step phase stepping. Under this condition the phase-stepping series must be completed within the sample rotation which corresponds to a single projection in tomography. On the other hand, G2 is moved step by step with a fixed step. Since there is no need to consider backlash problems as in the stepping motor stage, G2 is repetitively moved as shown in Fig. 2(*b*) ▶. The acquisition timing of the detector was controlled by the external trigger signals. The +5 V TTL trigger signals were also controlled by USB-6211. Actual signals of the input voltage and trigger signals during phase contrast tomography are shown in Fig. 2(*c*) ▶. The interval between the trigger signals corresponds to the sum of the exposure time, readout time of the camera (less than 50 ms) and delay of G2 movement (∼30 ms). The rotational speed of the samples should be controlled to perform a phase stepping properly under the scanning conditions shown in Fig. 2 ▶. Since a rotational stage (RA05; Kohzu Precision) is driven by a stepping motor with a resolution of 0.002° per pulse, it is easy to control the speed, which is expressed in pulses per second. The rotational speed is determined in the following manner. The number of pulses to rotate a sample for single projection in tomography is represented as *X*_proj_ = *X*_total_/*Y*, where *X*_total_ is the number of pulses to rotate the sample by 180° and *Y* is the number of projections. Then the rotational speed is defined as *X*_proj_/*T* (pulses s^−1^) where the time required for a phase-stepping series is assumed to be *T*. The time *T* is equal to *N*_step_ × (exposure time + readout time + delay) where *N*_step_ is the number of phase steps. Needless to say, a fast-readout camera is essential to achieve the scanning procedure shown in Fig. 2 ▶. Here a scientific CMOS camera with a frame rate of 100 Hz at full-frame readout (ORCA Flash4.0) was used.

The image quality in X-ray phase contrast tomography can be improved by acquiring direct images for flat-field correction at a fixed interval during the measurement. The interval can be changed according to the measurement conditions.

## 3. Application to a fresh sample   

### 3.1. Measurement of a fresh sample using X-ray phase contrast tomography   

High-throughput X-ray phase contrast tomography was applied to a fresh sample. As a fresh sample, mouse fetuses were used. Just after euthanasia of a pregnant mouse (BALB/c, Japan SLC), fetuses covered with a uterus membrane were removed by Caesarean section. The fresh mouse fetuses were put into a normal saline (Otsuka normal saline, NaCl 4.5 g/500 ml) and preserved in a refrigerator for approximately 2 h until measurement. Just before the measurement, the mouse fetus was embedded in a 2% agarose gel. The agarose block containing the mouse fetus was attached to a metallic rod using superglue and set on the sample stage. The temperature of the saline in the water cell was almost equal to room temperature (∼298 K).

The measurement conditions for the fresh mouse fetus were as follows. Number of projections: 600, with five-step phase stepping; interval for flat-field correction: 36°; exposure time: 500 ms per image. Under these conditions the rotational speed of the sample was calculated to be 51 pulses s^−1^. Since the rotational stage needs 90000 pulses for a 180° rotation, the total measurement time was approximately 30 min.

**Figure 3:**
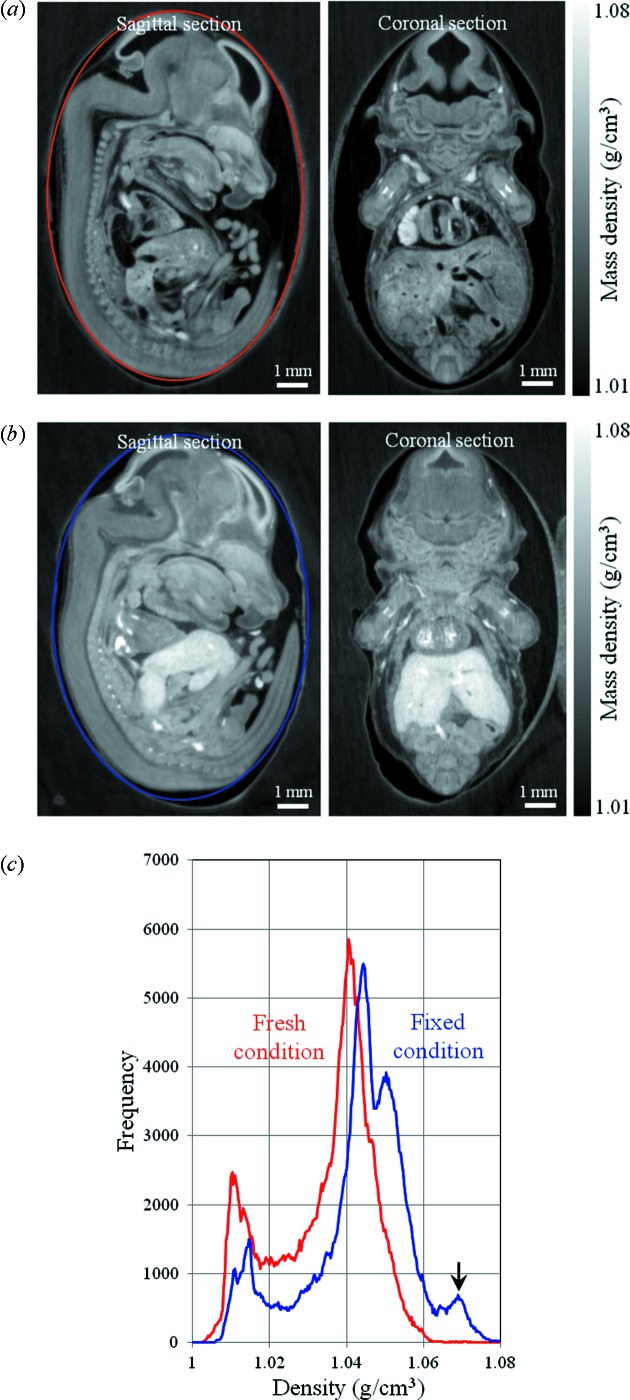
Phase contrast sectional images of (*a*) fresh and (*b*) formalin-fixed mouse fetus at gestation day 14. Left: sagittal section. Right: coronal section. (*c*) Histograms of the mass density measured at the ellipsoidal region of interest on the sagittal section.

Phase contrast sectional images of a fresh mouse fetus with 14 days of pregnancy are shown in Fig. 3(*a*) ▶. The sectional images represent sagittal and coronal planes of the mouse fetus. The gray scale of the sectional images is represented as mass density. Usually the reconstructed value in X-ray phase contrast tomography is represented as the difference in the refractive index Δδ. The relation between the phase factor δ and the mass density ρ is represented by the following equation,
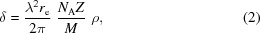
where λ is the X-ray wavelength, *r*_e_ is the classical electron radius, *N*_A_ is Avogadro’s constant, and *Z* and *M* are the number of electrons (atomic number) and molecular weight of the sample, respectively. So the difference in the density Δρ can be estimated from Δδ. Then the mass density of the sample can be estimated as ρ = ρ_w_ + Δρ where ρ_w_ is the mass density of the medium around the sample. The density map shown in Fig. 3(*a*) ▶ was estimated from Δδ in X-ray phase contrast tomography. In this estimation the *Z*/*M* value was assumed to be 0.55, which is an average for soft tissues (NIST database, http://physics.nist.gov/PhysRefData/XrayMassCoef/tab2.html). Δδ values were calibrated from standard samples (Hoshino *et al.*, 2010 ▶). In this case experimentally obtained Δδ values were multiplied by 1.45, the calibration factor under these measurement conditions. The calibration factor depends mainly on the X-ray detector.

Although the measurement time was approximately 30 min, motion artifacts associated with deformation or degeneration of the fresh tissue were not observed in the current spatial resolution. However, some motion artifacts were seen in the reconstructed image when the measurement took longer than 30 min.

### 3.2. Direct comparison between fresh and fixed samples   

The sectional images shown in Fig. 3(*a*) ▶ represent an original density map which is not affected by any fixatives. A mouse fetus measured in the fresh condition was carefully extracted from the agarose block and put into the formalin solution (10% formalin neutral buffer solution; Wako) to measure in the fixed condition. The difference between the fresh and fixed samples was directly compared to evaluate the effect of fixation on the soft tissue quantitatively. The fixation time was approximately 20 h at room temperature. The fixed mouse fetus was embedded in the 2% agarose gel again and measured under the same conditions. Phase contrast sectional images of the fixed mouse fetus are shown in Fig. 3(*b*) ▶. The image contrast was adjusted so as to be the same gray scale as for the fresh condition. Compared with the fresh condition, the mass density in the fixed sample became higher with formalin fixation. In particular, there are remarkable changes of density and volume in the liver. Histograms of the mass density measured at the ellipsoidal region of interest on the sagittal plane are shown in Fig. 3(*c*) ▶. The distribution of the density in the fixed condition shifted to a higher region. A new peak associated with the change of density in the liver (indicated by the arrow) was also generated at around 1.07 g cm^−3^ in the fixed condition.

**Figure 4:**
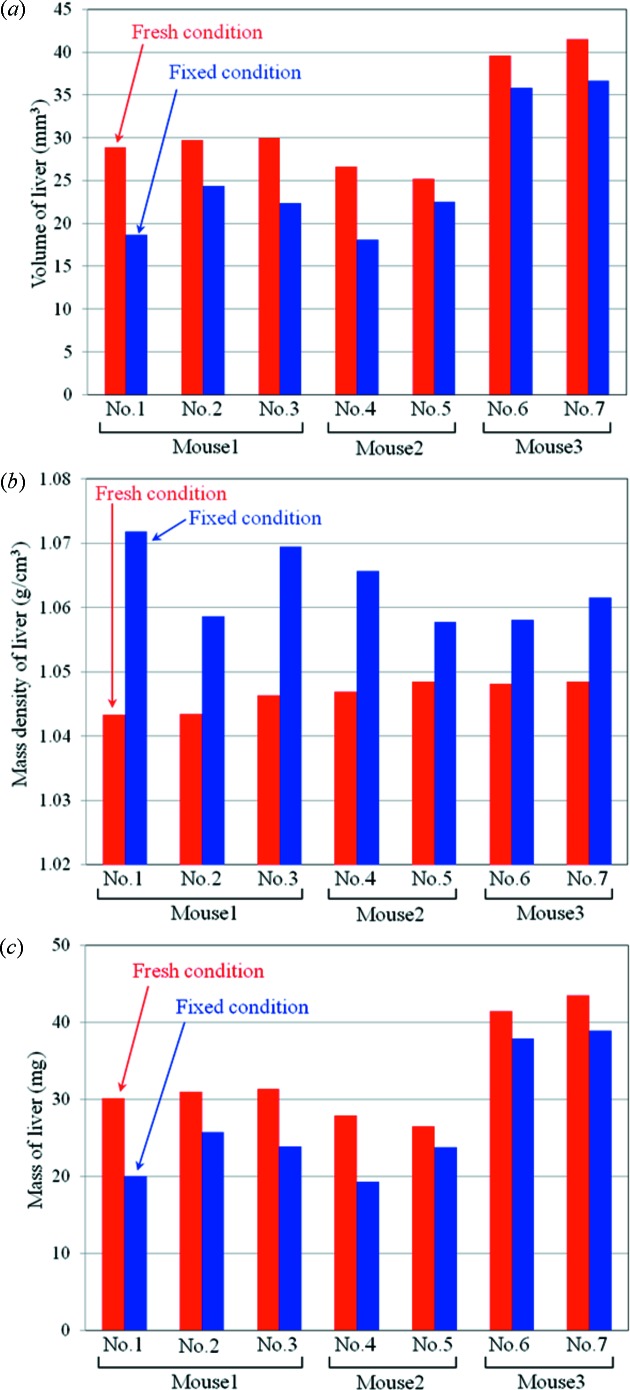
(*a*) Volume, (*b*) mass density and (*c*) mass of liver estimated from seven different mouse fetuses. The mouse fetuses used were randomly selected from three pregnant mice (mouse 1: 14 days of pregnancy; mouse 2: 14 days of pregnancy; mouse 3: 15 days of pregnancy).

As a quantitative evaluation between the fresh and fixed conditions, the volume and mass density of the liver were estimated from X-ray phase contrast tomography. To enhance statistical information, seven different mouse fetuses were measured under fresh and fixed conditions. Segmentation of the liver was performed manually using the image processing software *imageJ*. The mass density was estimated as an averaged value at an arbitrary region of interest in the liver. To estimate the mass density, the *Z*/*M* value was also assumed to be 0.55. The mouse fetuses used in this evaluation were obtained from three pregnant mice (two mice at 14 days of pregnancy, one mouse at 15 days of pregnancy). Two or three fetuses were randomly selected from each pregnant mouse. After measurements in the fresh condition, they were fixed by formalin for approximately 20 h and measured in the fixed condition. Results of the quantitative evaluation are shown in Fig. 4 ▶. Fig. 4(*a*) ▶ presents comparisons of the volume of the liver, and shows that the formalin fixation reduces the volume of the liver. The mouse fetus shown in Fig. 3 ▶ corresponds to specimen No. 1 in Fig. 4 ▶, which has the maximum shrinkage. In this case the ratio of shrinkage is approximately 35%. Fig. 4(*b*) ▶ shows that formalin fixation increases the mass density of the liver. In this case the increment of the density is 0.01–0.02 g cm^−3^. Fig. 4(*c*) ▶ shows the mass estimated from the volume and the mass density. The mass of the liver tends to be lower after the formalin fixation.

In most situations biological samples are fixed by formalin or other fixatives. Fixing a soft tissue is useful in order to preserve it for a long time. However, significant reduction of the volume and increment of the mass density in the liver have been confirmed in the formalin-fixed sample by directly comparing the phase contrast tomography images. Shrinkage of biological organs by formalin fixation has been studied for a long time. Recently, Schulz *et al.* reported a quantitative measurement of the shrinkage and strains field in human brain caused by formalin fixation based on MRI analysis (Schulz *et al.*, 2011 ▶). It is possible to evaluate the change of volume caused by fixation using three-dimensional imaging techniques such as MRI and X-ray microtomography. On the other hand, X-ray phase contrast tomography makes it possible to demonstrate the three-dimensional densitometry along with volume analysis. A small change in the density could be depicted as that in the apparent image contrast by the phase contrast imaging technique whereas these small changes could not be clearly visualized by simple absorption contrast imaging. Only X-ray phase contrast tomography enables the evaluation for such small changes of density quantitatively.

In the result shown in Fig. 4(*a*) ▶, the shrinkage of the liver was evaluated by manual segmentation, since there was a significant change in the volume. However, it is difficult to evaluate the small shrinkage or local strains from manual segmentation. In the case of strict evaluation of the change of volume and quantitative analysis of the strains field caused by fixation, the three-dimensional registration technique would be a powerful tool (Germann *et al.*, 2008 ▶; Schulz *et al.*, 2010*b* ▶; Bormann *et al.*, 2014 ▶).

## 4. From a static measurement to a dynamic measurement   

The high-throughput system shown above enabled measurement of fine phase contrast tomographic images and its application to fresh samples. This result might be a breakthrough in the application of X-ray phase contrast tomography to quantitative dynamic measurements of samples. In this study a preliminary measurement aiming at high-sensitive and quantitative imaging of a fresh sample accompanying a morphological change was demonstrated.

A ring-shaped fresh pig aorta was used for the measurement. The aorta is the largest blood vessel in which blood flow pumps out from the heart and passes through. It is exposed to blood pressure and constantly subjected to physical loads. Sometimes it suffers from fatal diseases such as aorta dissection. Understanding the morphological changes of the aorta under loaded conditions may be useful in clarifying the mechanism of the disease. However, it is difficult to observe the aorta *in vivo* with high image contrast using X-ray phase contrast tomography. Instead of an *in vivo* measurement, an extracted aorta was measured in the stretching condition to emulate *in situ* conditions.

**Figure 5:**
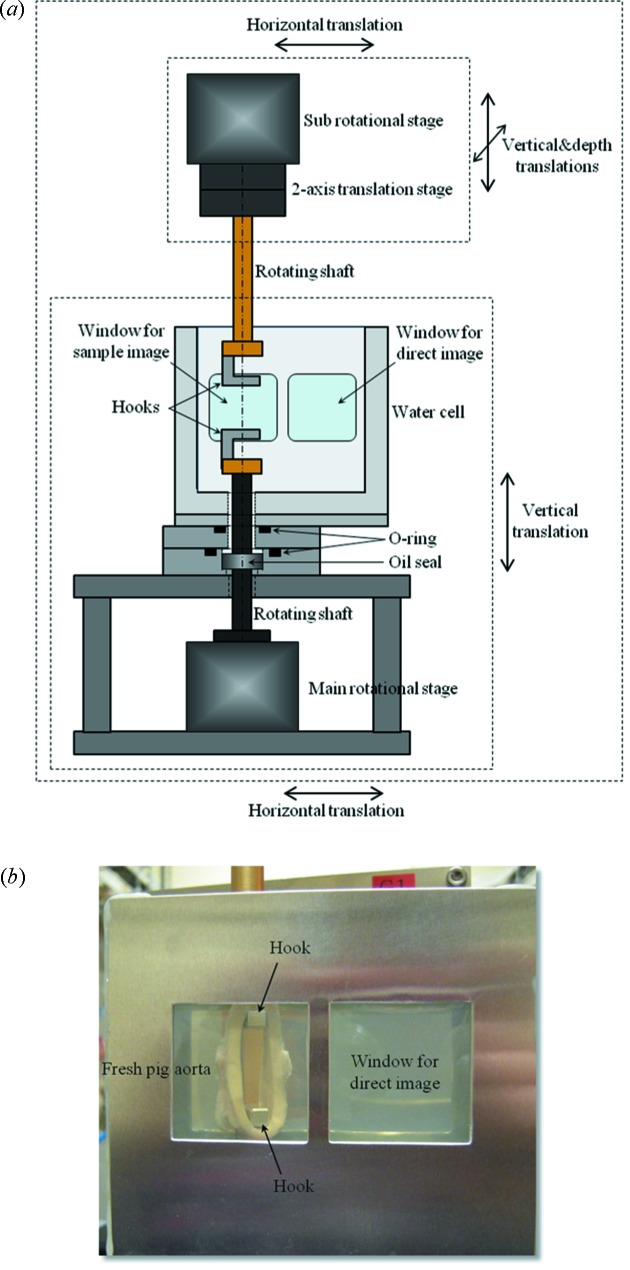
(*a*) Schematic drawing of X-ray phase contrast tomography for dynamic measurement of fresh pig aorta under stretching. (*b*) Photograph of a ring-shaped fresh pig aorta set on the sample stage.

A sample stage was modified to hold and stretch a ring-shaped aorta. In the previous system a sample in the agarose block was suspended by a rotational stage as mentioned above. To stretch the sample in the vertical direction, the main rotational stage was moved to below the water cell. A schematic drawing of the new sample stage is shown in Fig. 5(*a*) ▶. An oil seal was used to prevent leakage of liquid from the water cell to the rotational stage. Another rotational stage was installed above the water cell as a ‘sub-stage’ to stretch and rotate the sample. By driving the two rotational stages synchronously it became possible to rotate the sample under stretching. The ‘main’ and ‘sub’ rotational stages were placed on the same translation stage. Thus, the whole equipment could be moved horizontally for data acquisition of direct images used for flat-field correction. In this case the X-ray beam position was adjusted to the ‘Window for direct image’. A fine adjustment of the rotational axis between the ‘main’ and ‘sub’ rotational stages was made by moving the ‘sub’ rotational stage independently. The water cell was filled with normal saline as in the previous measurement. The thickness of the water cell was 25 mm.

A ring-shaped fresh pig aorta was cut out so that the width of the ring was approximately 8 mm. The fresh aorta was put in the saline and preserved in the refrigerator until measurement. The diameter of the aorta without any pressure was approximately 25 mm. Metal hooks were attached to the rotating shafts to hold the ring-shaped aorta. Fig. 5(*b*) ▶ shows the ring-shaped fresh pig aorta set on the sample stage. A small tension was loaded on the aorta in the initial state to prevent it from dropping from the hooks during measurement.

**Figure 6:**
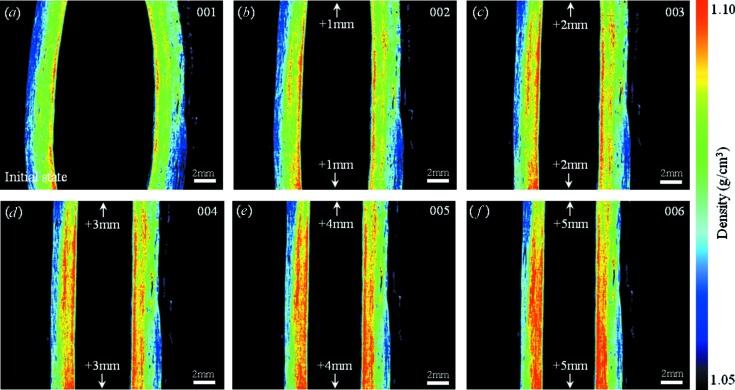
X-ray phase contrast computed tomography images of the ring-shaped fresh pig aorta in the stretching condition. (*a*) Initial state. (*b*)–(*f*) Stretched by hooks every 2 mm (1 mm by the upper hook and 1 mm by the lower hook).

**Figure 7:**
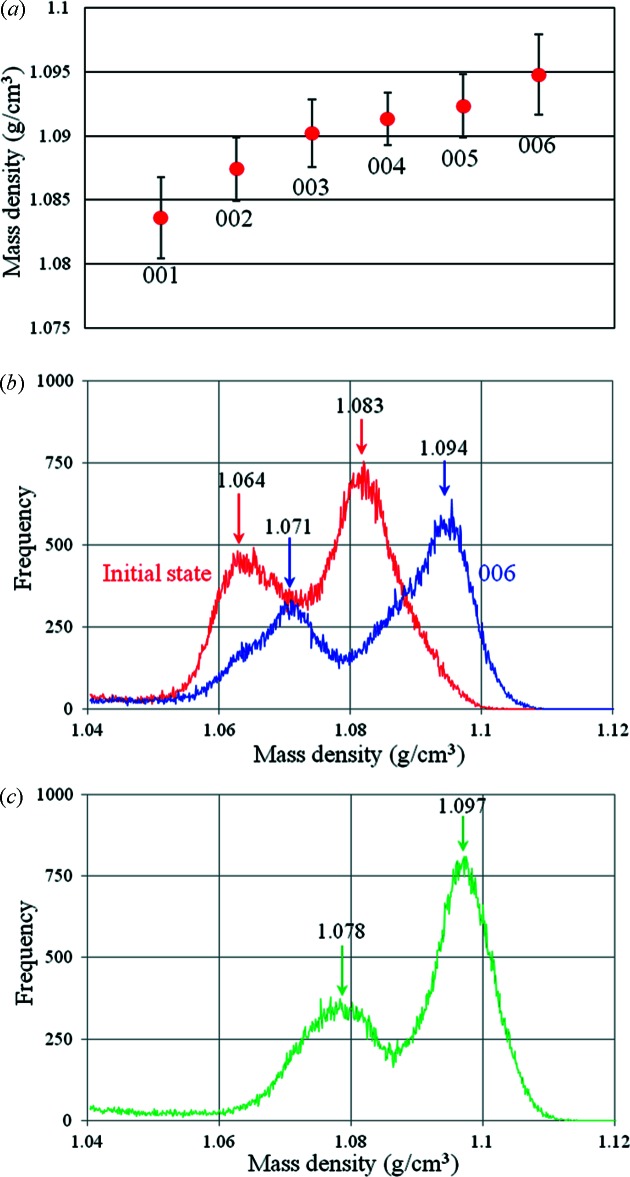
(*a*) Mass density measured at each step. The averaged value was estimated from the region of interest of 20 × 20 pixels on the sectional image. The error bars show the standard deviation. (*b*) Histograms of mass density measured at the left part of the sectional images shown in Figs. 6(*a*) and 6(*f*) ▶. (*c*) Histogram of mass density measured at the sectional image of formalin-fixed aorta.

The conditions for measurement of the fresh pig aorta were as follows. Number of projections: 600, with a five-step phase stepping; interval for flat-field correction: 18°; exposure time: 500 ms per image. Under these conditions the total measurement time was 38 min. The middle part of the aorta between the hooks was imaged. A phase contrast sectional image of the fresh pig aorta at its initial length is shown in Fig. 6(*a*) ▶. The sectional plane represents a vertical section. To demonstrate a dynamic measurement the aorta was stretched by hooks step by step. The amount of the stretch was 2 mm per step (1 mm by the upper hook and 1 mm by the lower hook). Phase contrast sectional images at each step are shown in Figs. 6(*b*)–6(*f*) ▶. The image contrast of the sectional images shown in Fig. 6 ▶ was adjusted by the same gray scale (expressed as mass density). By stretching the ring-shaped aorta the wall became thinner and the mass density at the inner side of the ring became higher. To evaluate the morphological change quantitatively, the mass density was estimated at each step. The result is shown in Fig. 7(*a*) ▶. In this case the mass density was defined as an averaged value in the region of interest (20 × 20 pixels on the image). Error bars represent the standard deviation. The region of interest used for the measurement was set at almost the same position with different degrees of stretch. Although the increment of the density was less than 0.005 g cm^−3^, it was clearly depicted as the difference in apparent image contrast by X-ray phase contrast tomography. Fig. 7(*b*) ▶ shows histograms of the density map at the initial state (Fig. 6*a* ▶) and the final state (Fig. 6*f* ▶). There are two peaks arising from the different densities in the tunica externa and tunica intima, and these two peaks shift towards higher density with stretching.

After measurements in the fresh condition, the aorta was fixed and measured in the fixed condition. Only a small tension was applied to the aorta. A histogram of the density map at the fixed condition is shown in Fig. 7(*c*) ▶. Although a large morphological change was not observed after the formalin fixation, the whole density became higher as was the case with the mouse fetuses described above.

## 5. Discussion   

A high-throughput measurement system in X-ray phase contrast tomography was introduced for application to biological fresh samples. Although it was difficult to directly compare the image quality with the previous system, the image quality in the current system would be sufficient to apply to high-contrast imaging of soft tissues. The density resolution of the reconstructed image estimated from the standard deviation of the background is 1.51 mg cm^−3^. This is almost the same as that in the previous system.

Some applications would require a quicker measurement time in X-ray phase contrast tomography. In the scanning procedure shown in Fig. 2 ▶, the measurement time directly depends on the exposure time and the number of phase steps when the number of projections is fixed. A measurement time of approximately 10 min was achieved with the five-step phase stepping by setting the exposure time to 100 ms per image. However, noise on the reconstructed image such as ring artifacts became more significant with shorter exposure times. The image quality was also affected by poorer X-ray photon statistics.

**Figure 8:**
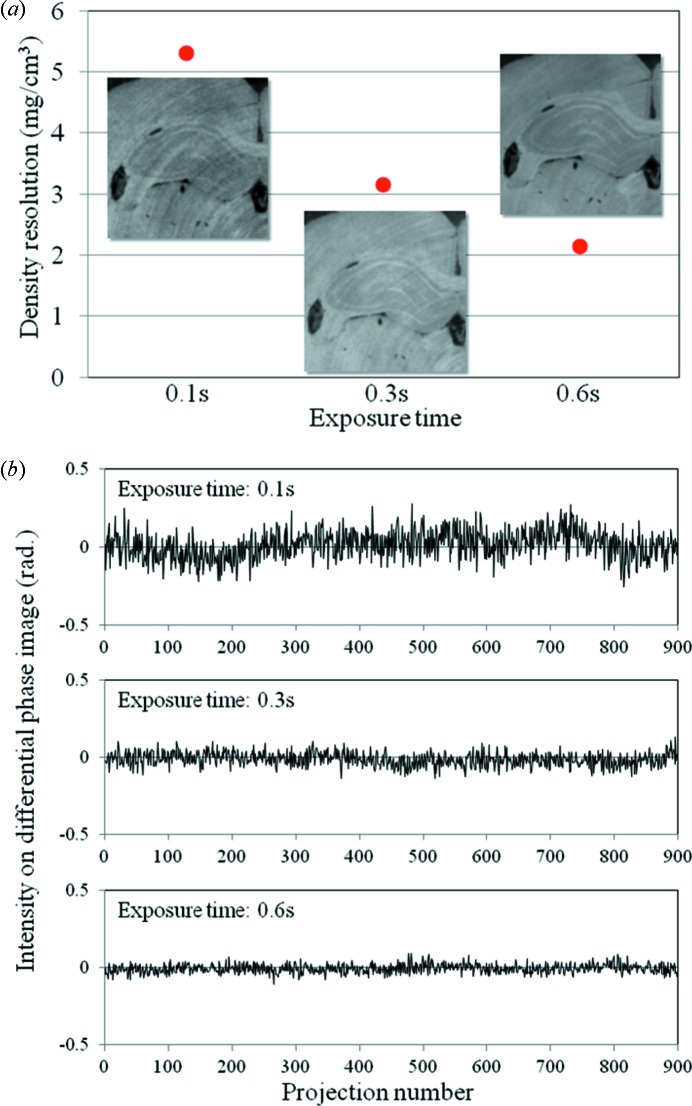
(*a*) Density resolution of sectional images obtained from X-ray phase contrast tomography with different exposure times. Insets represent a coronal plane of the hippocampus on rat brain at each exposure time. In this case the rotational axis is perpendicular to the coronal plane of the brain. The measurement conditions in X-ray phase contrast tomography are as follows: number of projections: 900, with five-step phase stepping; interval for flat-field correction: 18°. The effective pixel size is 24.6 µm per pixel (2 × 2 binning). In this condition the number of projections is approximately 1.5 times larger than the sampling width (the number of pixels) for the longitudinal direction in the coronal section of the rat brain. This means that the reconstructed images are over-sampled by projection images. (*b*) Fluctuations of the intensity in the background in the differential phase images during the tomographic measurement. The horizontal axis represents the number of projections.

To evaluate the image quality with the short exposure, the density resolution of the reconstructed images was examined. The density resolution with different exposure times is shown in Fig. 8(*a*) ▶. The density resolution becomes worse with a shorter exposure time due to poorer statistics of the signals that contribute to the image formation. The insets in Fig. 8(*a*) ▶ represent sectional images of the hippocampus of a formalin-fixed rat brain at each exposure time. In this case the number of projections seems to be sufficient against the sample size, namely the reconstructed image of the rat brain is over-sampled by the projection images. However, the ring artifact becomes stronger in the shorter exposure time. In the case of the shortest exposure condition in Fig. 8(*a*) ▶, the averaged signal in a phase-stepping series was almost 10% of the full-well capacity on the camera which had a 16-bit analog/digital (A/D) converter. Since the differential phase contrast image obtained from the phase stepping is based on five images, the amount of accumulated signal and corresponding statistical information seems to be sufficient to obtain fine reconstructed images. From this point of view the ring artifact in the reconstructed image might be caused by fluctuation of X-ray photons while a certain signal was obtained on the camera by a high conversion gain from X-ray photons to digitalized signals in the X-ray detector used. Fig. 8(*b*) ▶ shows the fluctuation of the intensity during the tomographic measurement for three exposure times. The intensity of a single pixel was measured in the background (without the sample) of the differential phase images. In this case, flat-field corrections for the differential phase images were performed for each condition. The high-frequency noise in Fig. 8(*b*) ▶ is due to poor X-ray photon statistics. With the shorter exposure time, a non-periodic fluctuation is apparent in the intensity. This fluctuation may affect the reconstructed image as the ring artifact.

The photon flux density of the monochromatic X-ray beam at the sample position is 1.2 × 10^8^ photons s^−1^ mm^−2^ with an X-ray energy of 25 keV. The photon flux at the detector position is reduced by the components in the system, especially the water cell. The number of X-ray photons which contribute to a single pixel on the X-ray detector is estimated to be approximately 860 photons in the projection image with an exposure time of 100 ms by considering the transmission of the system. Since more than 6000 A/D converted counts are detected on the camera, as described above, a single X-ray photon produces multiple A/D converted counts. Therefore, the digitalized signals on the camera are significantly influenced by the fluctuation of the X-ray photons. This situation is regarded as a photon-limited condition where one X-ray photon has a great influence on the image formation. In this case the fluctuation could be settled by increasing the exposure time as shown in Fig. 8(*b*) ▶.

To avoid the fluctuation of the X-ray photon flux with short exposure times, a high-brilliance X-ray beam obtained from an insertion device such as an undulator source is preferable. In the case of the standard undulator source at SPring-8, the photon flux density is of the order of 10^13^ photons s^−1^ mm^−2^ even in the monochromatic beam. This is five orders of magnitude larger than that in the current system which employs a bending magnet source. The high-brilliance X-ray beam would fix the issues of the fluctuation of the X-ray photons. In addition, it is possible to reduce the exposure time to less than 1 ms if the spatial resolution of the detector is set to be the same value as the current system. However, the beam size in the undulator source is quite small compared with the sample size targeted in this system. Although it is possible to expand the beam by using bent mirrors (Uesugi *et al.*, 2006 ▶), the spatial coherence which is necessary for phase contrast imaging must be considered in the reflected beam.

On the other hand, the measurement time will be drastically reduced by using white beam from a bending magnet source while keeping a moderate beam size. In this report a quantitative dynamic measurement of a fresh pig aorta was demonstrated using a ‘step-by-step’ stretching method. A real-time dynamic measurement would be possible by introducing a high photon flux beam. Actually the three-dimensional data of the sample could be obtained with a sub-second time scale in the X-ray tomographic measurement based on the simple absorption contrast (Mokso *et al.*, 2011 ▶; Xiao *et al.*, 2012 ▶; Hoshino *et al.*, 2013 ▶). Since the grating interferometry works well with a polychromatic beam, real-time measurements in X-ray phase contrast tomography have also been reported (Momose *et al.*, 2011 ▶; Olbinado *et al.*, 2013 ▶). In those cases, ultra-fast phase contrast imaging was achieved by using white-beam synchrotron radiation. However, radiation damage when using white-beam radiation would be unavoidable. Overdose will destroy soft tissues and make it difficult to obtain correct information from the sample. Furthermore, in most applications using X-ray phase contrast tomography, a histological study with an optical microscope is performed after the X-ray phase contrast tomography, for which a damaged sample is no longer useful. From this perspective on the biomedical application it is important to consider the optimum condition to obtain the largest amount of information from the sample even in the dynamic measurement.

**Figure 9:**
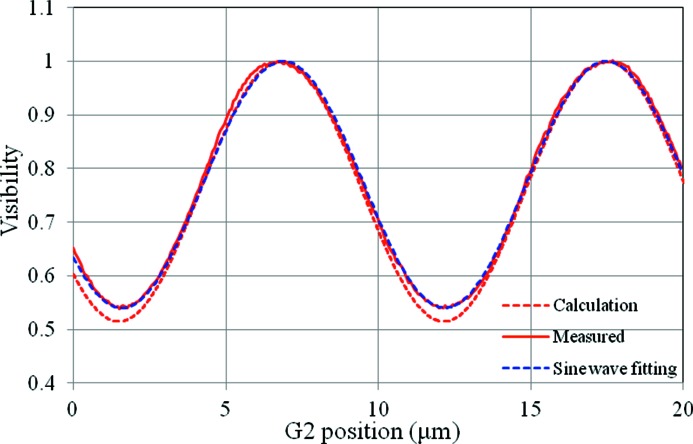
Measured (red solid line) and calculated (red broken line) visibility profiles in the current system. Sine-wave fitting for the measured visibility is also shown as a blue broken line.

Reduction of the number of steps in the phase stepping is one of the approaches used to shorten the measurement time (Uesugi *et al.*, 2011*b* ▶). The measurement time would be three-fifths of the current system by changing from five steps to three steps. The three-step measurement is the minimum case for phase retrieval using phase stepping. In this case the shape of the visibility profile must be considered. In general it is represented as a triangle profile under coherent illumination. With a partial coherent source like synchrotron radiation, the visibility profile becomes close to a sine wave. The number of steps in the phase stepping can be effectively reduced if the visibility profile is represented as a sine-like wave. The visibility profile in the current system is shown in Fig. 9 ▶. The solid line and broken line represent measured and calculated visibility profiles, respectively. In the calculation the source size and the spatial coherence of the X-ray beam are considered. The measured visibility profile can be regarded as almost a sine wave. Thus, the number of steps in the phase stepping may be replaced from five to three in the current setup.

## 6. Conclusion   

X-ray phase contrast tomography was successfully applied to quantitative measurements of fresh samples based on densitometric analysis. High-throughput X-ray phase contrast tomography with a measurement time of 30 min could be used to measure the small density differences in the fresh samples with adequate density resolution. The small change in the density between the fresh and formalin-fixed samples could also be depicted as apparent image contrast by X-ray phase contrast tomography. This is a prominent advantage compared with other three-dimensional imaging techniques. The morphological change of the soft tissue caused by a mechanical load could also be captured as a small increment of the mass density using the proposed method. This three-dimensional densitometry can be a powerful tool for revealing the native density map of biological soft tissues which has a great significance in their biological functions. Furthermore, this technique will be strengthened by combining with the quantitative three-dimensional registration technique for analysing the small change of volume in soft tissues. X-ray phase contrast tomography and subsequent three-dimensional densitometry using synchrotron radiation will be a promising technique for biomedical imaging which requires a high resolving power for soft tissue densities in static and dynamic states.
